# Genomics in plant pathogen identification and control

**DOI:** 10.3389/fpls.2025.1661432

**Published:** 2025-11-05

**Authors:** Nkechi Gloria Ogbuji, Josephine Udunma Agogbua

**Affiliations:** Department of Plant Science and Biotechnology, University of Port Harcourt, Port Harcourt, Nigeria

**Keywords:** plant-pathogen interaction, genomic technologies, NGS, CRISPR-Cas9, RNAi, metagenomics, disease-resistant crops, sustainable plant protection

## Abstract

Recent advances in genomics have revolutionized plant pathogen detection and control by enabling faster and more accurate identification compared to traditional culture-based methods. Genomic tools like metagenomics and next generation sequencing (NGS) facilitate the detection of microorganisms (bacteria, fungi, viruses, and nematodes) directly from environmental samples. Genomics also provides information on plant-pathogen interactions, especially the detection of Resistance (R) genes and their role in plant defense against pathogens, aiding in the development of genetic markers for breeding disease-resistance crop species. Gene editing systems such as clustered regularly interspaced short palindromic repeats (CRISPR) associated protein 9 (CRISPR-Cas9), transcription activator-like effector nucleases (TALENs), and Zinc Finger nucleases (ZFNs) allow for precise genetic modification, either by incorporating a beneficial R genes or disabling susceptibility (S) genes of the host plant. RNA interference (RNAi) is another genomic tool used to suppress important pathogenic genes and inhibit disease development. Although the use of genomics in plant pathology is hampered by limitations such as high costs, complexity of data analysis and interpretation, and limited access to sequencing platforms, especially in developing countries, recent innovations and multi-disciplinary collaborations are tackling these issues. In general, genomics offers powerful tools that can be employed in the development of sustainable and effective plant disease management strategies, which will help to enhance crop protection and contribute to global food security.

## 1 Introduction

The increasing global population has intensified the demand for food, both in terms of quantity and quality. Plant diseases pose a major threat to agricultural productivity, often resulting in significant yield losses, reduced crop quality, and loss of biodiversity. These impacts, in turn, have detrimental socio-economic and environmental consequences (Singh et al., 2023; Gai and Wang, 2024). Climate change further raises the risks of outbreaks by changing pathogen evolution and host-pathogen interactions, and promoting the development of new stains of pathogens (Singh et al., 2023). Effective plant disease management hinges on the early detection and accurate identification of pathogens, alongside an in-depth understanding of pathogen virulence mechanisms and host-pathogen interactions (Gai and Wang, 2024; Hamim et al., 2024). Early identification of the causal agents of disease enables timely intervention and the implementation of appropriate management strategies, thereby minimizing losses (Khakimov et al., 2022). While traditional diagnostic techniques—such as culture-based methods and morphological analyses—have long been used, they are often time-consuming, labor-intensive, and lack sensitivity and specificity. These methods are typically unable to identify pathogens at the species or strain level and rely heavily on expert interpretation of symptoms, which may only appear during specific plant growth stages. Although fully asymptomatic plants do not show loss in yield, they can act as a source of inoculums to spread the pathogen to other plants that are susceptible.

The field of plant pathology has transitioned from conventional methods to the genomic era, driven by advancements in molecular biology and computational tools. Notably, next-generation sequencing (NGS)—also known as high-throughput sequencing (HTS)—has enabled comprehensive genome and transcriptome analyses. HTS is a technology that allows for the sequencing of several DNA samples in a single run (Tamang, 2024). The sequencing speed and efficiency of HTS is higher than that of traditional sequencing methods. It facilitates the identification of resistance genes and helps researchers to determine how genes are expressed during pathogenic attacks (Malook et al., 2024). NGS enhances disease management by detecting novel pathogens, tracking disease outbreaks, and supporting the development of resistant plant varieties (Nizamani et al., 2023). It also allows the identification of non-cultivable and emerging pathogens directly from plant microbiomes (Abhishek et al., 2025). For example, Abdelrazek et al. (2025) utilized culture-independent, long-read metagenomic sequencing of DNA from wilt-affected tomato (*Lycopersicon esculentum*) to achieve strain-level resolution and predict resistance and virulence genes using bioinformatics platforms.

Biological information generated from host-pathogen interactions provide researchers with an improved understanding of plant health and agricultural productivity. Our understanding of plant-pathogen interactions has been significantly increased through genomics researches by revealing the mechanisms of pathogen virulence and host plant defense. Pathogenic microorganisms, including bacteria employ virulence factors and effectors to evade or repress plant immune responses and efficiently colonize host tissues (Anderson, 2023; Asif et al., 2024). Resistance (*R*) genes are genes in plants that confer disease resistance against pathogens by producing proteins called R proteins while susceptibility (*S*) genes are genes exploited by pathogens to aid invasion and penetration of host plants. In plants, pattern recognition receptors (PRRs) located on cell surfaces detect pathogen effectors or molecules released during pathogen invasion. This initiates the primary defense mechanism in plants, referred to as pattern-triggered immunity (PTI) (Nguyen et al., 2021). Nucleotide‐binding/leucine‐rich repeat (NLR) receptors inside the host cells detect pathogen effectors which activates effector‐triggered immunity (ETI), a more powerful and long-lasting defense response (Hou et al., 2019; Yu et al., 2024). More than 213 typical *R* genes that confer resistance to various diseases have been discovered in wheat, barley rice, maize, and other crop species (Kourelis and van der Hoorn, 2018; Li et al., 2020). Through genome-wide association studies the *R* gene *LABR_64* and the partial resistance gene *PiPR1* were identified in rice (Kang et al., 2016; Liu et al., 2020).

The CRISPR-Cas systems have been used for genome editing, enhancing our understanding of disease resistance mechanisms and assisting researchers to improve the function of *R* gene and disrupt *S* genes, offering new pathways to developing lasting resistance (Malook et al., 2024; Pandarinathan et al., 2024). *S* genes are frequently utilized by plant pathogens to enhance their growth and facilitate infection. The disruption of *S* genes creates opportunities for breeding of resistance crops species. The engineering of *S* genes in genomes using CRISPR-Cas system has been reported in many crops of agriculturally importance (Bishnoi et al., 2023). Proteins involved in important physiological functions such as defense response, pathogen detection and signal transduction are encoded by *S* genes. New techniques to speed up *R* gene cloning have been developed as a result of breakthroughs in genome sequencing and bioinformatics (Wulff and Moscou, 2014).

In addition to genome editing, RNA interference (RNAi) offers a non-transgenic approach for disease control by silencing essential pathogen genes. RNAi employs double-stranded RNA to target and degrade specific mRNA sequences, effectively inhibiting gene expression (Banik et al., 2025). This method has shown promising results in plant disease control, especially in cases where control options are limited, making RNAi a valuable gene-based therapy for plant protection (Pandarinathan et al., 2024).

This comprehensive review explores the transformative impact of cutting-edge genomic tools on the detection, characterization, and management of plant pathogens, showcasing the latest advancements that are revolutionizing plant pathology. Furthermore, it critically examines emerging genome-driven disease control strategies and addresses existing technical and practical challenges, offering insightful perspectives to guide future research and facilitate the broader adoption of genomics in sustainable plant disease management.

## 2 Genomics in detection and identification of plant pathogens

Early detection and accurate identification of pathogenic microorganisms is necessary for the development of appropriate control measures (Comtet et al., 2015). Mechanisms of plant-pathogen interactions, host specificity, identification of causative agents of diseases have all been studied using genomics (Xu and Wang, 2019). Formerly, plant pathogens were identified using traditional method which combines the use of visual symptoms, microscopy and culturing of microbes. Since traditional method of pathogen identification largely relies on prior knowledge of microbes, they may lead to wrong identification because of the similar morphologic and microscopic structures of pathogens, and similar patterns of disease symptoms (Nezhad, 2014). Although these methods are cheap but culturing of microorganisms is hectic and time consuming and many microbes cannot be cultured on any known growth media. Less than 1% of the total microorganisms on earth have been described to date (Schultz et al., 2023), with the help of genomics tools, more microbial species on earth can be detected, identified and described. New and efficient methods that allow for rapid detection and identification of pathogens, both known and cryptic species, without the need of cultivating or culturing these pathogens have been developed (Bard et al., 2024). Advances in Plant pathology gave rise to the combination of traditional methods with fast and reliable molecular methods, such as polymerase chain reaction (PCR), in the identification of plant pathogens. Genetically similar pathogens which are difficult to differentiate by PCR can be distinguished using next-generation sequencing (NGS) technologies which produce data that can provide information on an organism’s whole genome, single nucleotide polymorphisms (SNPs), and small sequence repeat (SSR) (Withers et al., 2016; Pecman et al., 2017). The genomic sequences of non-culturable pathogens can be generated through genome or metagenome sequencing using NGS technology, which is rapid, cheap and accurate. Genomic sequences provide taxonomic and genomic information that provide the basis for the identification of microorganisms and also provide information on the basic function of pathogenic or virulent genes which can aid in the development of new disease diagnostic methods (Xu and Wang, 2019).

Genomic analysis has been useful in describing Liberacter species since most species of Liberacter cannot be cultured. “*Candidatus* Liberibacter asiaticus” (CLas) is an alpha-proteobacteria that causes citrus huanglongbing, a devastating disease currently threatening global citrus industry (Zheng et al., 2024). The major challenge researchers face in working with Ca. Liberibacter is that it inhabits the phloem, a complicated environment that cannot be easily manipulated (Pandey et al., 2021). Phloem-limited pathogens are a group of plant pathogens that primarily infect and reside within the phloem tissue of plants, causing significant economic losses in agriculture. The sensitivity, rapidity, and relatively cheap nature of NGS have made the use of genome sequencing for the detection and identification of emerging pathogens possible (Xu and Wang, 2019). Through comparative genomics analyses, evolutionary history pathogenecity and host-specific adaptations of Liberibacter genus can be determined and genome regions associated with pathogenicity have been identified (Batarseh et al., 2023; Zheng et al., 2024). This is achieved by analyzing genome sequences from different species or strains to identify genetic variables associated with pathogenicity and adaptation. Through NGS, Díaz-Cruz et al. (2019) identified nine foliar pathogens in *Glycine max* (soybean) plants, which were not previously reported in Manitoba province, Canada. The pathogens are: six fungi (*Alternaria tenuissima, Cercospora sojina, Colletotrichum gloeosporioides, Colletotrichum graminicola, Diaporthe eres and Pleospora herbarum*), two bacteria (*Pseudomonas cichorii and Pseudomonas syringae* pv. tabaci), and a virus, *Bean yellow mosaic virus*. NGS successfully identified the pathovars of some of the pathogens and assembled complete or near complete genome sequences of the RNA viruses that were identified. The information obtained from the study was used to develop a PCR-based diagnostic method for seven of the most prevalent pathogens identified in the province. This will encourage quick, reliable, and cost-effective pathogen identification in the area.

### 2.1 Genome sequencing

A genome is the complete set of all the genetic material in an organism. It contains the DNA and genes with their coding and non-coding regions (Xiong et al., 2023). Genome sequencing is the determination of the complete DNA sequences present in an organism, providing complete genetic information of the organism. The advent of NGS has made it easier to sequence several millions of DNA fragments at a time, improving research in plant disease diagnosis and control (Iovino, 2022; Satam et al., 2023). The genetic diversity of species, loci of genetic variations, adaptative mechanisms among other important genetic information can be detected through genome sequencing (Gao et al., 2024). Analysis of DNA through genome sequencing using NGS can be carried out in different ways such as whole-genome sequencing, whole-exome sequencing, and targeted sequencing. A summary and comparison of the various approaches through which NGS-based genome sequencing can be performed is presented in **Table 1**.

**Table 1:** Summary and comparison of various genome sequencing platforms.

Characteristic	Whole genome sequencing	Whole exome sequencing	Targeted sequencing
Focus of sequencing	Entire genome (all genes of the organism)	Exome (protein-coding regions)	Gene of interest (selected loci or regions)
Purpose of sequencing	Detection of variations	Identification of phenotypic variations	Diagnosis
Variants that can be detected	InDels, CNVs, SNPs, and DNA methylation	InDels, CNVs, and SNPs,	Gene duplications, rearrangements, small insertions and deletions, and SNPs
Data obtained	Huge data requiring complex bioinformatics platform for analysis	Moderate data	Relatively small data that can be easily analyzed
Sequencing technology	Illumine, Nanopore MinION, PacBio	Illumina, Capture-based TS	Illumina, Amplicon-based TS, and Capture-based TS

#### 2.1.1 Whole genome sequencing

WGS provides an in-depth view of an organism’s complete genetic composition and is particularly effective for detecting genomic variations across different species (Lu et al., 2025). It has been used in gene identification, functional predictions and association of certain genes with disease traits. The advancement of WGS has been largely driven by next-generation sequencing (NGS) technologies, which generate massive sequence data subsequently analyzed using advanced computational and bioinformatics tools (Yin et al., 2019). By aligning reads to reference genomes, genetic variants such as Single Nucleotide Polymorphisms (SNPs), Copy Number Variations (CNVs), and Insertions and Deletions (InDels), can be identified, providing detailed genetic data for differentiating species and strains of organisms (Davey et al., 2011; Fuentes-Pardo and Ruzzante, 2017; Wu et al., 2019). Through WGS, DNA methylation, a vital epigenetic modification that plays a pivotal role in cellular processes and gene regulation, can be identified (Wreczycka et al., 2017). Although early research implied that DNA methylation suppresses gene expression, an increasing amount of data suggests that, depending on the genomic region where DNA methylation occurs, DNA methylation can play both an inhibitory and a permissive role (Jones, 2012). DNA methylation can indirectly regulate gene expression by affecting the accessibility of chromatin for transcription factors or by engaging repressive proteins with methyl-binding domains (Cedar and Bergman, 2009). DNA methylation patterns can be altered rapidly upon exposure of cells to changing environments and pathogens (Qin et al., 2021). The expression of important genes involved in immune responses in plants can be altered as a result of alteration in DNA methylation and/or regulation of the expression and function of DNA methylation modifiers such as DNMTs (DNA methyltransferases) and TETs (ten-eleven translocation proteins) (Pacis et al., 2015). These changes in DNA methylation may help pathogens to evade the host immune system and remain within the host, or they may help protect host immunity to eradicate pathogens. Cultivable isolates of bacteria and fungi can be easily and rapidly identified and other information such as susceptibility to antimicrobials, and disease outbreak investigation and surveillance, can be revealed through WGS (Ko et al., 2022; Nwadiugwu and Monteiro, 2022).

#### 2.1.2 Whole-exome sequencing

**Figure 1:**
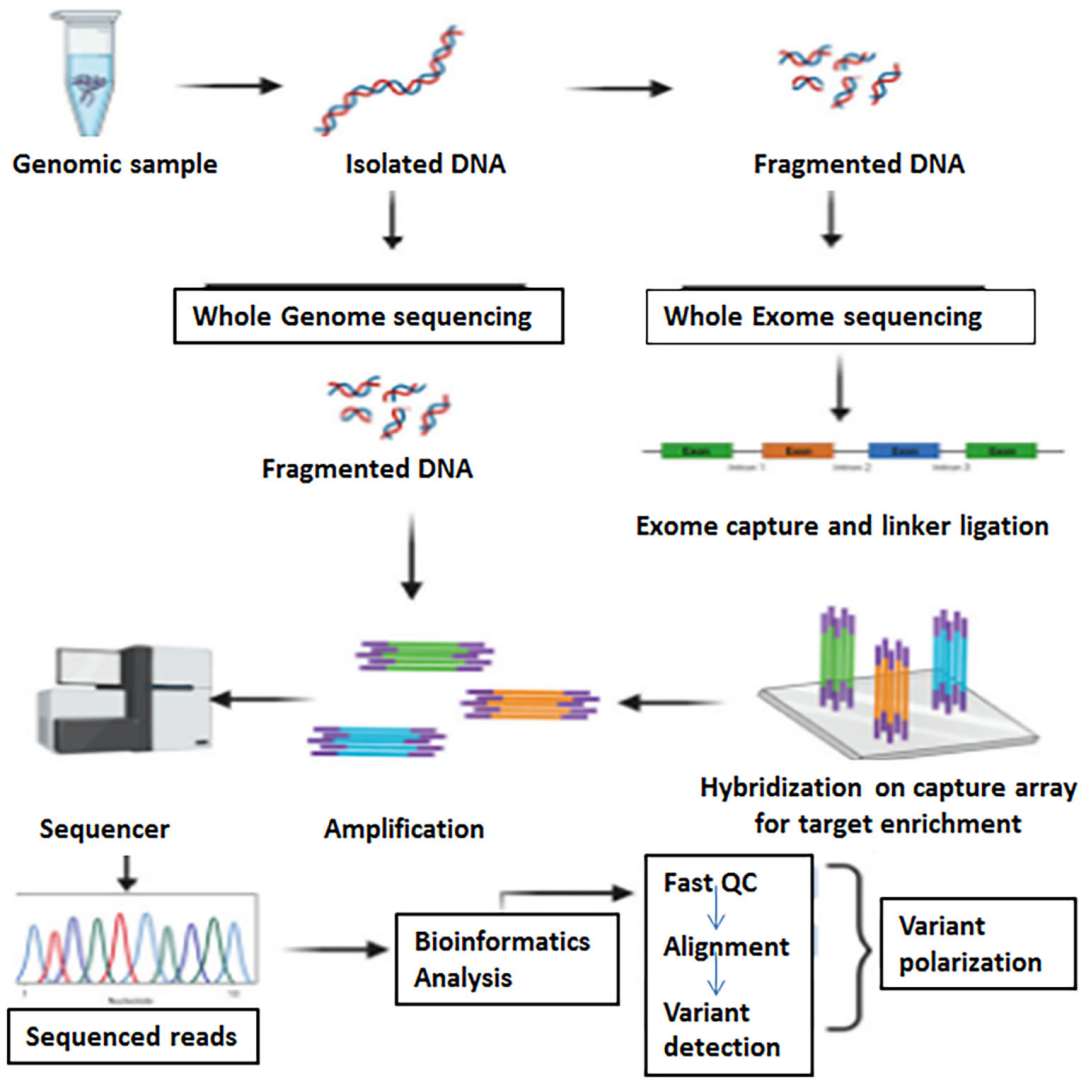
A schematic representation of whole genome and whole exome sequencing workflow (Adapted from Prasad et al., 2021).

Whole-exome sequencing (WES) focuses on the sequencing of the protein-coding regions of the genome, called the exome. About 1 to 2% of the entire genome is made up of exomes which contains most of the known disease-causing variants (Satam et al., 2023). WES allows for the identification of genetic variations, such as insertions, deletions, CNVs, and SNVs, within protein-coding genes (Rabbani et al., 2014; Logsdon et al., 2020). An in-depth WGS is costly; WES is a cost-effective substitute to WGS and a direct approach for detecting phenotype-associated variants in protein-coding regions of genomes (Bogaerts et al., 2025). WES entails the enrichment of exons through target-specific amplification or hybrid capture methods, followed by NGS. A schematic representation of Whole Genome and Whole Exome Sequencing workflow is presented in **Figure 1**.

#### 2.1.3 Targeted sequencing

TS focuses on the specific regions of a gene. It allows scientists to select and analyze loci of interest, producing huge genomic data at these desired regions, and reducing labor and sequencing costs. It is capable of picking up different types of genetic variations such as SNVs, small gene insertions, deletions, duplications, or rearrangements associated with diseases (Gilpatrick et al., 2020). TS is an important technique in both research and clinical settings, with high accuracy and rapid turnaround time. The technique focuses on the amplification of a target gene or sequences of interest, therefore allowing for specificity in the detection of sequence variations which is crucial in disease diagnosis (Paskey et al., 2019). The target genes or regions of interest are usually related to pathogenesis of diseases (Pei et al., 2023). The quantity and quality of extracted nucleic acid (DNA/RNA) such as the concentration, purity (OD 260/280 ratio), and fragment size are determinant factors for choosing the reagents and approaches that would be used for TS (Petersen and Coleman, 2020). The two methods of TS commonly used are amplicon and capture-based approaches. The amplicon approach uses a pre-designed specific primer to amplify the target regions before the library preparation (Bewicke-Copley et al., 2019). The capture-based approach involves the fragmentation of DNA and the hybridization of oligonucleotide attached to sequence-specific RNA or DNA probes to capture the region of interest (Gaudin and Desnues, 2018; Paskey et al., 2019).

### 2.2 Metagenomics as a tool for identification of plant pathogens

Metagenomics is a culture-independent approach used to directly analyze prokaryotic genome such as fungi, bacteria, and viruses present in a sample (Thomas et al., 2012) either by amplicon (or targeted) sequencing or by shotgun sequencing (Tedersoo et al., 2015). The highly conserved regions and hyper variable regions in 18S or 16S ribosomal RNA genes of fungi and bacteria is used for the amplicon sequencing, and this aids in the identification of each organism present in the sample. For fungal species characterization, the internal transcribed spacer regions 1 and 2 (ITS1 and ITS2) covering the 5.8S rRNA gene of the fungal genome are amplified (Bellemain et al., 2010). For bacterial organisms, identification is based on the amplification of the hyper variable (V) regions V1 to V2 or V3 to V4 (Walker et al., 2020). Targeted sequencing includes the following steps: sample collection and optimization, DNA extraction, selection of appropriate barcode primers, PCR amplification, library preparation, and high thorough-put sequencing carried on either short reads or long reads platform (Aragona et al., 2022). The resulting sequences are analyzed using different bioinformatics pipelines. Low quality sequences are filtered out and high quality sequences are clustered into operational taxonomic units (OTUs) at 97% similarity or amplicon sequence variants (ASVs). OTUs or ASVs are compared to sequences on databases to identify the microbes (Cao et al., 2017). OTUs are taxonomically classified and their functions predicted. The use of amplicon sequencing for the detection of phyto-pathogens has been reported by many researchers (Piombo et al., 2021). Amplicon sequencing of the full length ITS region using PacBio sequencing led to the detection of fungal organisms in soil samples, with species-level classification (Tedersoo et al., 2018). Another study used Nanopore MinION for amplicon sequencing to detect and identify *Xylella* species in leaves, with a sub-species level resolution. Pathogen detection was obtained within 15 minutes of sequencing (Marcolungo et al., 2022). Strain level identification for *Xylella fastidiosa* was obtained through amplicon sequencing using MLSA marker genes (Faino et al., 2021).

Shotgun metagenomics allows for the sequencing of the entire genome of microbes from environmental samples such as soil, symptomatic and asymptomatic host plants, providing an effective method of identification of pathogens (MechanLlontop et al., 2020). Shotgun metagenomics avoids PCR-associated biases and generate data from longer DNA regions, thus producing more reliable results. However, it also provides more genomic information about the pathogenic and other metabolic characteristics of the organisms in the sample (Venbrux et al., 2023). Shotgun metagenomics has the capacity to detect all the phyto-pathogens and possibly new ones present in a sample (Piombo et al., 2021). Third-generation sequencing technologies such as Oxford Nanopore MinION have revolutionized shotgun metagenomics, making it more accessible, affordable and less time-consuming. This technology is widely used for sequencing viral RNA/DNA, plant viral disease surveillance, viral genome assembly, and evolutionary relationship of viral particles (Sun et al., 2022a). Yang et al. (2022) was able to distinguish the blight fungal pathogen of boxwood (*Calonectria pseudonaviculata*) from *Calonectria henricotiae* using Nanopore MinION. Metagenomic data can be used to obtain the necessary information required for the identification of the microbes present in a sample or give more understanding of the functional genes of the microorganisms (Lapidus and Korobeynikov, 2021; Semenov, 2021).

### 2.3 Bioinformatics tools for genomics data analysis

In culture-dependent genomic studies, sequences obtained through conventional molecular techniques such as DNA extraction, polymerase chain reaction (PCR) and Sanger sequencing are processed and analyzed using simple bioinformatics tools such as MEGA X, Bio-Edit etc. These tools facilitate sequence editing, alignment and comparison with publicly available databases such as, National Center for Biotechnology Information (NCBI), allowing for species identification based on sequence similarity. NGS instruments generate huge amounts of RNA or DNA sequence data. Handling, processing, analyzing, and interpreting these data to obtain biological information require computational methods. Bioinformatics approaches which involve various computational methods, tools and algorithms, that handle pre-processing, alignment, gene expression quantification, and other specific analyses are used for analyzing NGS data (Satam et al., 2023). Modern plant pathology depends on bioinformatics to develop new plant disease diagnostic tools. Recent developments in genomics and molecular biology techniques have led to the successful generation of vast biological data. Host–pathogen genome data provides the avenue for retrieving, annotating, analyzing, and identifying sequences and denoting their functions for characterization at the gene and genome levels (Joshi et al., 2023). Once the sequences are processed, different computational techniques, such as *de novo* assembly, and reference-based mapping, are applied to obtain the required biological information. Bioinformatics tools also facilitate the identification of genetic variations, such as SNPs, CNVs, and SVs (structural variants). The combination of NGS data with other genomic and functional data, enable the understanding of gene expression and regulatory systems. Some bioinformatics tools used for different analysis of NGS data according to Satam et al. (2023) are listed below.

A. For common analysis

- Quality check of sequences (FASTX-toolkit, FastQC, MultiQC)
- Trimming of adaptors and low-quality bases (Trimmomatic, fastp, Cutadapt)
- Alignment of sequence reads to reference genome (dragMAP, Bowtie, BWA)
- Reports visualization (MultiQC)

B. For whole-genome sequencing, whole-exome sequencing and targeted sequencing

- Removal of duplicate reads (Sambamba, Picard)
- SNPs and indels (Platypus, GATK, DeepVariant, freeBayes, Illumina Dragen, VarScan)
- Filter and merge variants (bcftools)
- Variant annotation (NIRVANA, ANNOVAR, snpEff, ensemblVEP)
- Structural variant calling (Manta, DELLY, Pindel, GRIDDS, Lumpy, Wham)
- Copy number variation (CNV) calling (CNVnator, ExomeDepth for CNVs from Exome, cn.MOPS, GATKgCNV, cnvCapSeq for targeted sequencing)

C. For shotgun metagenomics

- Taxonomic classification (Kraken, MetaPhlAn4, Kaiju)
- Assembly of metagenomic reads (metaIDBA, metaSPAdes)
- Protein databases for taxonomic classification (NCBI non-redundant protein database)
- Gene annotation (MetaGeneMark, Prokka)
- Databases for functional annotation of genes (GO, KEGG, COG)

### 2.4 Sequencing technologies for identification of plant pathogens

#### 2.4.1 Sanger sequencing

Molecular identification of plant pathogens often begins with PCR amplification of targeted genetic regions from DNA extracted using culture-based method. There are various PCR techniques commonly used in the field of crop disease detection, including nested PCR, quantitative PCR, multiplex PCR, digital PCR, nanoparticle-assisted PCR, immuno PCR, reverse transcription PCR and other novel PCR (long PCR, GC-rich PCR, fast PCR, direct PCR, hot start PCR, touchdown PCR) (Zhang et al., 2025). The obtained PCR products or amplicons are then sequenced.

The real breakthrough in molecular biology came with the introduction of the chain termination-based sequencing method by Fredrick Sanger (Sanger et al., 1977). Sanger sequencing, also known as dideoxy sequencing, provided the foundation for DNA sequencing. It is a first generation sequencing technology that involves using deoxynucleotides (dNTPs) to synthesize a DNA strand that is complementary to the template. When DNA polymerase incorporates 2, 3′-dideoxynucleotides (ddNTPs), synthesis is terminated (Sanger et al., 1977). Fragments of various lengths are produced and separated by gel electrophoresis inside capillaries. Each of the four ddNTPs is tagged with a different fluorescent dye. As labeled fragments pass through the DNA sequencer, the dye is excited by a laser, and the resulting fluorescence emission of one of the four colors is used for base-calling and sequence assembly (Smith et al., 1986). The sequencing proceeds in parallel (Paegel et al., 2002) with an output capability of up to 2 million bases during a 24 h period.

Four hundred and seventy-eight microbial genomes have been sequenced using Sanger sequencing. This includes *Streptococcus pneumoniae* and *Bacillus anthracis* (Tettelin et al., 2001; Read et al., 2003) among others. These studies provided the information required for the development of methods for detecting gene locations and numbers, prediction of proteins, and pseudogenes. Moreover, insertion and deletion of sequences, and horizontally transferred genetic elements, such as plasmids and bacteriophages were identified through Sanger sequencing. Sequences were uploaded on databases, making them available to the public. With this, researchers can compare the genomes of related pathogens, allowing for a better understanding of pathogenesis and evolution of microorganisms. With advances in genomics, other sequencing technologies which are relatively cheaper, faster and more precise than Sanger sequencing have been developed.

#### 2.4.2 High thorough-put sequencing methods

High throughput sequencing or next-generation sequencing (NGS) represents various new and evolving technologies for sequencing of millions of DNA fragments simultaneously that basically vary in their ways of recording nucleotides. HTS platforms greatly differ in their read length, accuracy, volume of data produced, and cost (Tedersoo et al., 2021). Although sample preparation and analyses of NGS data can be technically demanding and require skilled personnel (Yu et al., 2021), it has proven to be cost-effective, rapid, and highly accurate, enabling simultaneous sequencing of millions of DNA fragments. This provides comprehensive insights into genome structure, gene function, and genetic variations.

NGS technologies are categorized into short-read sequencing platforms (e.g., 454 pyrosequencing, Illumina, Ion Torrent), and long-read sequencing platforms (e.g., Pacific Biosciences [PacBio] and Oxford Nanopore [ONT]) (Levy and Myers, 2016). These technologies have significantly enhanced the detection and identification of pathogens, including novel and emerging pathogens, development of improved cultivars, and genome editing (Klosterman et al., 2016; Silva et al., 2019; Saeed et al., 2023). NGS has played a significant role in plant disease management, disease surveillance and pathogen evolution, which are vital for developing effective disease management strategies (Van der Heyden et al., 2021; Kawasaki et al., 2023). With recent developments in NGS technology, traditional culturing method is gradually being replaced by HTS methods like Ion torrent and Illumina sequencing for the identification of fungi, viruses, and bacteria (Saeed et al., 2023). In Plant Pathology, NGS has been successfully used to detect pathogens in diseased plants, asymptomatic plants and in plants that don’t show specific symptoms (Jones et al., 2017; Keremane et al., 2024). For effective application of NGS, strong bioinformatics resources and workflows are required to analyze the data obtained. Such assignments as pathogens classification and host sequence removal from reads can be successfully carried out on these workflows (Jones et al., 2017).

454 pyrosequencing (Roche Diagnostics, Basel, Switzerland) was the first NGS instrument to be developed, and was launched in early 2000s. Pyrosequencing is a first-generation sequencing technology. The technology was more than 100 times cheaper (10^−2^ EUR/read) than Sanger sequencing, and has a read length of 50 to 700 -1,000 base pairs (bp) at 1.2 million read throughput (Reuter et al., 2015). 454 has been used to identify parasitic nematodes (Porazinska et al., 2009). The technology is based on a strategy of “single-nucleotide addition”, which depends on a sole signal to record the addition of a dNTP into an elongating strand. To ensure that the signal is brought about by only one dNTP, each of the four nucleotides must be separately added to the sequencing reaction. 454 pyrosequencing is now obsolete due to high costs and limited scalability (Reuter et al., 2015; Aragona et al., 2022).

Ion Torrent (www.thermofisher.com/ng/en/home/brands/ion-torrent) and Illumina (www.illumina.com) technologies, introduced in early 2010s, replaced 454 because they generate more data and are relatively cheaper. These two platforms are referred to as second-generation sequencing technologies. However, limitations in the use of Ion Torrent exists as it produces short read length (about 450 bp) and has fluctuating sequence quality. This is why it cannot be used to sequence DNA from plant and soil samples (Kemler et al., 2013). Illumina next-generation sequencing technology provides about 3,000 times greater throughput than the 454 technology. It has a read length of up to 300 base pairs (bp) and an accuracy rate of >99.5% (Goodwin et al., 2016). It is relatively cheaper (10^−5^ to 10^−4^ EUR/read), more accurate, and has the likelihood to sequence reads of up to 550 bp (2 × 300 paired-end). With Illumina, more than 1000 samples can be analyzed in a single run at a high sequencing depth (Zinger et al., 2017). Illumina uses terminator molecules (dNTPs) in which the ribose 3′-OH group is blocked, to prevent elongation. Incorporation of a single dNTP to each elongating complementary strand is followed by recording the image of the surface in order to identify which dNTP was added at each cluster. To start a new cycle, the blocking group and fluorophore are removed. Illumina is the most common and widely used short-read sequencing because of its sequence quality, cost-effectiveness (Knief, 2014), high level of cross-platform compatibility, and its wide range of platforms. Cline et al. (2017) reported the increase in the relative abundance of soil pathogens with increases in plant biomass in grassland using Illumina NGS. Bainard et al. (2017) through Illumina NGS, detected that crop rotation using leguminous crops highly increases the abundance of pathogens in the soil.

**Figure 2:**
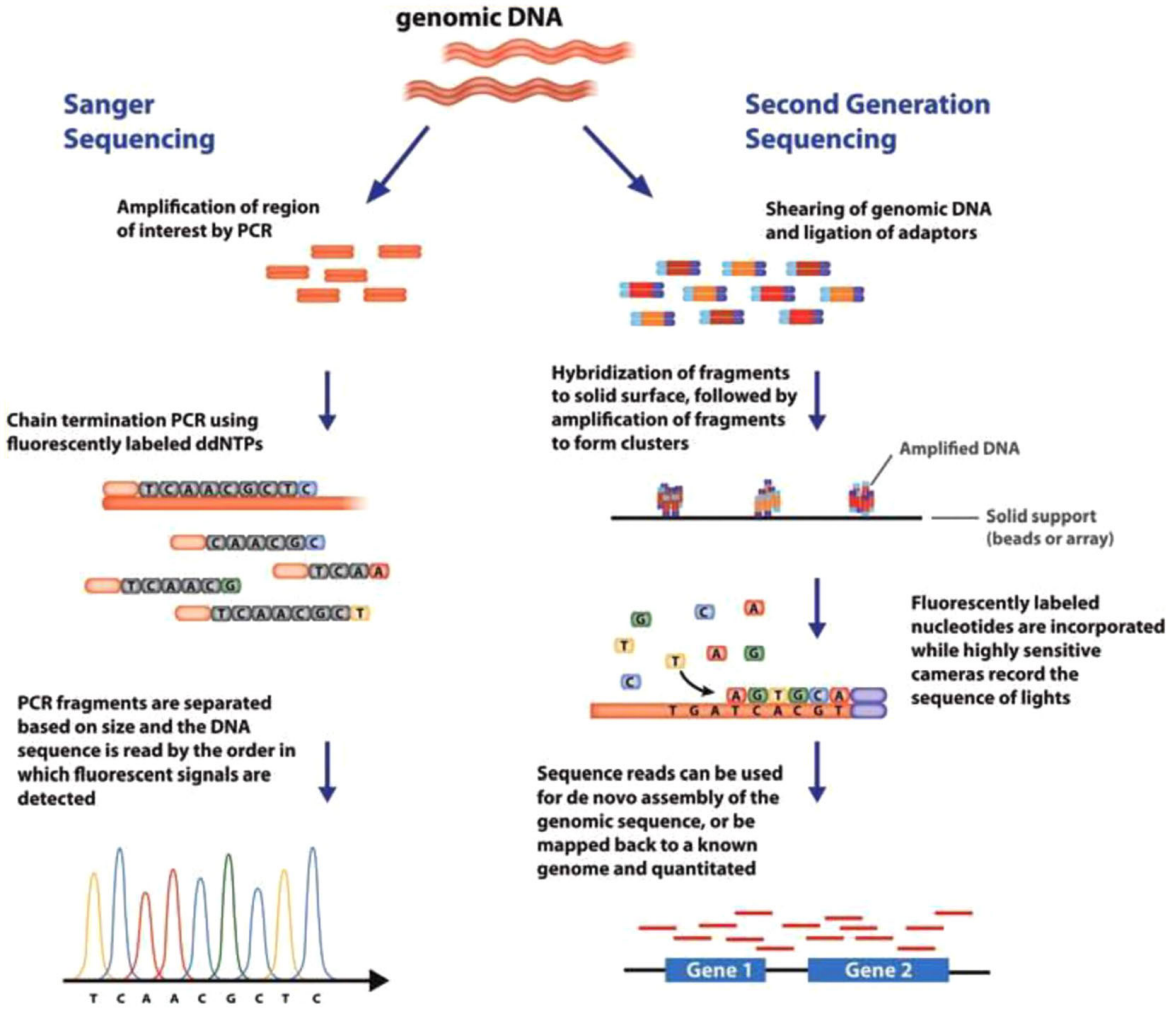
Comparison between Sanger sequencing and next-generation sequencing (NGS) technologies (Adopted from Bunnik and Le Roch, 2013).

All the sequencing technologies discussed above produce short reads. Sequencing platforms that generate long reads have been developed and are termed third generation sequencing technologies. DNA fragments containing tens of kilobases can be sequenced on these platforms. Short-read sequencing involves sequencing by synthesis based on enrichment of samples through fragmentation, amplification, or hybridization while long read sequencing basically depends on sequence detection either by synthesis or by electrical voltage change/resistivity, producing current when a single base passes through the biological membrane pore. Short read sequencing can generate reads between 600 to 700 bp whereas long read sequencing can generate about 25 to 30 kb read length. Furthermore, the amplification bias is eliminated in long-read sequencing as opposed to short-read sequencing. Since the library preparation in long-read sequencing technologies excludes PCR, DNA methylation and other base modifications can be easily detected (Satam et al., 2023). Third-generation sequencing platforms include Oxford Nanopore Technologies (ONT, UK), Single-molecule real-time (SMRT) sequencing by Pacific Biosciences (PacBio, USA), and the Helicos™ Genetic Analysis System by SeqLL (LLC, USA) (Ambardar et al., 2016). PacBio and ONT were commercialized in 2011 and 2015, respectively. The SMRT platform can sequence single molecules. It uses hairpin adaptors to form a closed single stranded DNA (ssDNA) template (SMRT bell) which is placed in a zeptoliter-sized compartment, with a sole polymerase particle at the bottom of the compartment. The addition of fluorescently-labeled nucleotides in the phosphate group is detected in real-time (Rhoads and Au, 2015). PacBio sequencing is relatively more expensive than Illumina sequencing, costing about 300 EUR/library and 10^−2^ EUR/read. Oxford Nanopore MinION sequencing is based on nanopore technology. DNA sequences are determined by changes in electrical current which occurs when a single-stranded DNA molecule passes through a nanopore. It transforms base-specific fluctuations into DNA sequences as a result of a nanopore blockage (Lu et al., 2016). This technology is popular because of its portability and simplicity. MinION pocket-sized device is the most popular device for real-time whole genome sequencing and sequencing of DNA/RNA sequences (Tyler et al., 2018). It has been used for identification of strains, cryptic species and WGS analyses, as in the case of *Escherichia coli* (Loman et al., 2015). Frey et al. (2022) developed a rapid and accurate sequencing tool that provides genetic information at different taxonomical levels. The workflow was designed such that a prior knowledge of the target organism, such as specific primers, primer binding sites and/or its classification is not required. The workflow also made provision for the collection of genetic data encompassing the whole genome, allowing the identification of species using single or multiple genetic markers that their sequence information can be established or readily available. They used whole genome amplification (WGA) and Oxford nanopore MinION sequencing to show the suitability of the workflow for the identification of two bacteria (*Escherichia coli* and *Erwinia amylovora*a), three fungi (*Neofabraea alba*, *Colletotrichum salicis* and *Cladosporium herbarum*), and a nematode, *Globodera rostochiensis*. Comparison between Sanger sequencing and next-generation sequencing (NGS) technologies is presented in **Figure 2**.

## 3 Understanding plant pathogen virulence factors and resistance mechanisms

In the face of climate change and the increasing demands of a growing global population, a comprehensive understanding of plant pathogens is critical for developing sustainable and effective disease management strategies. Such insights are essential for safeguarding crop productivity and ensuring global food security (Hamim et al., 2024). Genomic analysis of plant pathogens provides valuable information on their virulence factors and mechanisms of pathogenicity and host resistance, which are key components in devising targeted control strategies. Virulence factors are a diverse group of molecular and structural components produced by pathogenic microorganisms that enable them to evade host immune responses and establish infection successfully. This includes enzymes, toxins, exopolysaccharides, cell surface structures such as capsules, glycoproteins, lipoproteins, and lipopolysaccharides. It is also known that virulence is influenced by intracellular alterations in metabolic regulatory networks that are controlled by non-coding regulatory RNAs and protein sensors/regulators (Leitão, 2020). Phenotypes like virulence depend on genomic data. Therefore, to identify the genetic bases of traits such as virulence and resistance, genome sequences can be compared across isolates or species (Schikora-Tamarit and Gabaldón, 2022). The genomes of fungal pathogens are highly dynamic, which facilitate rapid adaptation to environmental changes. These genomic changes are often associated with the emergence or enhancement of virulence traits, allowing pathogens to overcome plant defense mechanisms more effectively (Ferrari et al., 2009).

### 3.1 Virulence factors

Virulence factors are the chemical or biochemical signals that pathogens employ to evade or suppress the host defense system in order to successfully infect and spread in the host (Cross, 2008). The sequencing of pathogen genomes provides necessary information for predicting infection strategies and identifying potential virulence factors. Functional prediction and analysis of virulence factors such as effector proteins, that interact with host resistance genes and contribute to pathogenicity, offer great insights into resistance durability and future resistance engineering of plants (Upadhyaya et al., 2021). Resistant durability is the period of time between the release (first use) of a resistant cultivar and the point at which the pathogen genotype overcomes resistance and invades the plant population, which basically signifies the breakdown of resistance (Lo Iacono et al., 2013). The time frame can range from a few years to decades. Information obtained from functional prediction and analysis of virulence factors can help breeders to choose and develop resistant genes that are less likely to be defeated by pathogen evolution, thus improving the longevity of resistance in crop varieties. This can be utilized to direct risk assessment and disease management plans, which will ultimately reduce the likelihood of future disease outbreaks and aid in preparing for new pathogen outbreaks (Aylward et al., 2017).

Parasitic plants are complex organisms, more complicated than microorganisms and pathogens. They possess specific proteins associated with virulence, and are considered pests because they cause diseases in host plants (Zhang et al., 2014). Functional classification of SNP to ascertain the roles of specific gene families, transcriptome sequencing, and genome annotation provide insight into the differences between virulence genes (pathogen effectors) and host resistance genes. The genome of the sunflower broomrape, *Orobanche cumana*, encodes 221 proteins with a leucine-rich repeat (LRR) domain (Hélène et al., 2017). In addition to LRR domains, *Striga hermonthica* secretome study revealed several cysteine-rich small proteins linked to cell wall modification and protease activities, and are also involved in *S. hermonthica-*host plant interaction (Hegenauer et al., 2016). Host plants also recognize and react to molecular signals released by parasitic plants in order to counter their virulence (Hu et al., 2020). For instance, Cuscuta Receptor 1 (CuRe1) is a surface receptor found in tomato plants that reacts to the peptide factor of *Cuscuta* spp. It triggers an immune response and recognizes parasitic plants in a way that is comparable to how microbial diseases are perceived (Bradley et al., 2019).

#### 3.1.1 Virulence factors of bacterial organisms

*Ralstonia solanacearum* is a well-established model for studying phyto-pathogenic bacteria because of its high genetic diversity, environmental persistence, and broad host range (Vailleau and Genin, 2023). It utilizes diverse virulence tools such as Type III effectors (T3Es) and extracellular enzymes to invade host tissues and suppress immunity (An and Zhang, 2024). *R. solanacearum* species complex consists of over 100 different T3Es. Depending on the strain, 50 to 75 type III effector (T3E) proteins pass through the type 3 secretion system (T3SS), and are translocated into plant cells (Landry et al., 2020). Reported strains contain 60 to 75 T3Es each (Sabbagh et al., 2019). Depending on the host plant, an effector can act in different ways, all gearing towards bringing down the host defense arsenal. For instance, the effector, *Ralstonia* injected protein AB (RipAB) has been reported to inhibit the activities of TGA transcription factors in Arabidopsis (Qi et al., 2022) and down-regulate calcium-signaling pathways in potato (Zheng et al., 2019). *R. solanacearum* also secretes exo-polygalacuturonases (PehC), a cell wall-degrading enzyme which prevents the activation of pattern-triggered immunity (PTI) in the host, and also provides essential carbon sources required by *R. solanacearum* for growth and multiplication during the onset of infection in the xylem, serving a unique dual-purpose virulence strategy (Laflamme, 2023).

Amylovoran is an exopolysaccharide (EPS) that plays a role in the pathogenicity of *Erwinia amylovora.* It has a high viscosity, which hinders the movement of water through the vascular system (Dellagi et al., 1998; Piqué et al., 2015). *E. amylovora* strains that do not produce amylovoran have been reported to be non-pathogenic (Piqué et al., 2015). Le van, a homopolymer made up of fructose residues is another virulence factor of *E. amylovora*. Levan and amylovoran have been reported to play a role in the production of bacterial biofilm in xylem vessels (Koczan et al., 2009). The attachment of bacterial organisms to surfaces is dependent on biofilm production. When iron supply is limited, *E. amylovora* produces siderophores such as desferrioxamines (DFO) that play dual role in pathogenicity (Dellagi et al., 1998).

*Xanthomonas* species release lipopolysaccharides (LPS), EPS, degradative enzymes, and adhesins to facilitate host infection (Büttner and Bonas, 2010). Xanthan, the major EPS contributes significantly to the pathogenicity of *Xanthomonas campestris* pv. campestris (Xcc), by inhibiting the deposition of callose in plant cell walls of *Arabidopsis thaliana* and *Nicotiana benthamiana*, and predisposing the plants to infection (Yun et al., 2006). LPS plays a role in the basal defense activation in host and non-host plants. LPS protect bacterial cell from unconducive environments. Additionally, *Xcc* forms biofilms composed of proteins, lipids and EPS, which strengthens the pathogen’s defense against external stimuli (Dow et al., 2003; Li et al., 2019a; Büttner and Bonas, 2010). Bacteria that cannot synthesize EPS are unable to form biofilms (Dow et al., 2003). The bacterium uses cyclic *β*-(1,2)-glucans to maintain homeostasis and further enhance virulence (Buonaurio, 2008). *Xanthomonas axonopodis* pv. citri (Xac) is one of the pathogens responsible for citrus canker. Xac mutations in the LPS biosynthesis genes (*wzt* and *rfb303*) and their impact on interactions with tobacco and orange plants were investigated by Petrocelli et al. (2012). Xac mutants displayed altered bacterial motilities and increased susceptibility to environmental stressors. Variations were noted in the expression levels of the genes involved in LPS production. In host plants, Xac*wzt* showed less virulence than Xac wild-type and Xac*rfb30*3. *Xanthomonas citri* ssp. *citri* possesses the non-fimbrial adhesin protein Xac*FhaB*, which is necessary for bacterial attachment and has been shown to be a significant virulence factor for the development of citrus canker (Garavaglia et al., 2016). Bacterial adherence to the host, biofilm development, and aggregation are all facilitated by Xac*FhaB*. In host and non-host plants, all adhesin regions were able to stimulate basal immune responses. *Candidatus* Liberibacter spp. employ various virulence mechanisms such as LPS, bacterial effectors (bacterial proteins produced in host cells), flagella that aid colonization and increase plant immunity, salicylic acid hydroxylase (an enzyme) and prophages. The LPS trigger plant immune responses, leading to callose deposition, phloem blockage, or programmed cell death (PCD), which interferes with nutrient transport and causes systemic symptoms (Wang et al., 2017; De Moraes Pontes et al., 2020).

#### 3.1.2 Virulence factors of fungal organisms

Fungal pathogens possess specialized structures which they use to completely invade plants by penetrating their organs. Plant pathogenic fungi that cause important diseases in agriculture contain virulence factor genes that perform different functions (Wang et al., 2022). Fungi virulence factors includes mycotoxins, effectors, organic acids and cell wall-degrading enzymes, which they use to invade plants and cause diseases. Virulence factors help fungi pathogens to suppress the host plant defense, extract nutrients, and have control over the living tissues of the host. Infection strategy vary among fungi: necrotrophs consume dead host tissues by secreting poisonous chemicals which kill their host’s cells; biotrophs do not cause direct death of the plant tissues, they feed on living tissues but the effector chemicals they release reduce the growth of the host cells and regulate host plant metabolism in their advantage; hemibiotrophs combine the infection patterns of both necrotrophs and biotrophs (Kumar et al., 2024).

*Rhizoctonia* species have complex infection modes and are resistant to standard fungicides, endangering the worlds food supply (Nizamani et al., 2025). Climate change affects pathogen dynamics and reduces the effectiveness of conventional management methods. *Rhizoctonia* spp. secret RsCRP1 effector that target host or plant mitochondria and chloroplasts, in order to suppress host, and RsSCR10, which induces plant cell death (Tzelepis et al., 2021; Niu et al., 2021). *Alternaria alternata* produces host-specific toxins (HSTs) like AM-toxin, which disrupts cell membranes in pear and apple plants (Tanahashi et al., 2016), and tentoxin, which causes chlorosis and spots in hosts by deactivating the protein involved in energy transmission on chloroplasts and preventing the phosphorylation of ADP (Binyamin et al., 2019). *Botrytis cinerea* produces the superoxide dismutase (SOD) BCSOD1enzyme on the host surface, which facilitates penetration by appressoria (specialized cells found in many fungal plant pathogens that is used to infect host plants). In multiple hosts, mutants lacking BCSOD1 show a decrease in pathogenicity (Rolke et al., 2004). *B*. *cinerea* also produces several phytotoxic proteins and secondary metabolites such as, botrydial, which causes chlorotic lesions in host plants (Colmenares et al., 2002). Beauvericin (BEA), Deoxynivalenol (DON), and fusaric acid are the metabolites that contribute to virulence of *Fusarium* species (Audenaert et al., 2013; López-Berges et al., 2013; López-Díaz et al., 2018). The genes *FfVel1*, *FfVel2* and *FfLae1* have been linked to virulence in *Fusarium* (Wiemann et al., 2010). DON inhibits DNA and proteins biosynthesis, causes apoptosis, programmed cell death (PCD) and peroxidation of lipids, and disrupts cell membrane. BEA aids infection, as shown by reduced pathogenecity following NRPS gene deletion (López-Berges et al., 2013). Although the role of fusaric acid in virulence mechanism remains unclear, it likely contributes to vascular wilt by affecting water permeability of plants (López-Berges et al., 2013; López-Díaz et al., 2018).

Generally, research on virulence factors has greatly advanced our understanding of plant-pathogen interactions, enabling insights into molecular basis of pathogenicity and improved knowledge of plant (De Moraes Pontes et al., 2020).

### 3.2 Comparative genomics detects virulence factors

Comparative genomics is the study of the evolutionary comparison of genomic sequences and gene content across species that differ in a particular trait (e.g. virulence towards a particular host). This approach allows researchers to develop hypothesis on the potential relationships of the species by detecting genomic changes such as duplication, loss, acceleration, or acquisition of certain genes that correlate with the presence or strength of the trait. With the increasing availability of whole genome sequences of pathogens and their close non-pathogenic relatives, comparative genomic studies are becoming more efficient (Schikora-Tamarit and Gabaldón, 2022). Comparative genomics has been effectively used by different researches to understand the development of virulence and resistance in various fungal pathogen lineages. One prominent example is *Fusarium oxysporum*, which may have developed into a widespread plant pathogen as a result of horizontal acquisition of virulence genes (Ma et al., 2010). Bertazzoni et al. (2018) reported that meiotically unstable ‘accessory chromosomes’, which encode genes linked to host specificity, are the primary cause of the pathogenic behavior of *F. oxysporum* sub-species.

Genomic variation within and across populations of a given species (population genomics) can also be studied. Once a reference genome is available, sequencing multiple individuals from different populations allows for the identification of single nucleotide polymorphisms (SNPs), deletions, insertions, and other variations. The frequency and spread of these variations can reveal the genetic make-up of a species, identify sub-clades, trace population history, reconstruct the history of populations, and pinpoint genomic regions subject to selection (Schikora-Tamarit and Gabaldón, 2022). A minor deletion in the *Zt_8_609* gene of the wheat plant pathogen, *Zymoseptoria tritici* is linked to virulence. This deletion is involved in a particular interaction with a plant resistance gene. The deletion is therefore essential for virulence (Hartmann et al., 2017). This further demonstrates how gene loss might influence significant fungal traits.

### 3.3 Plant pathogens resistance mechanisms

Development of resistance in plant pathogens threatens disease management in plant pathology. Pathogens develop mechanisms to survive and continually infect crops as a response to changes in the environment and chemical treatments. Understanding of pathogenic resistance mechanisms is necessary for the development of effective disease diagnosis and management tools. A good example is the emergence of fludioxonil-resistant strains of *Botrytis cinerea* which have made control of gray mold disease of cherry tomato crops more difficult (Liu et al., 2023b). This disease causes significant economic loss, although the fungicide, fludioxonil, has been proven to be effective in its control. However, some strains of *B. cinerea* have developed resistance to this fungicide. Liu et al. (2023a) employed RNA sequencing and quantitative real-time PCR (qRT-PCR) to discover the genes that play a role in fludioxonil resistance in *B. cinerea*. The authors identified sixteen fludioxonil-responsive genes among which nine were up-regulated while six were down-regulated. These genes included major facilitator super family (MFS) transporter-encoding genes, high-osmolarity glycerol (HOG) pathway homologues or related genes and adenosine triphosphate (ATP)-binding cassette (ABC) transporter-encoding genes. The findings from the study revealed the resistance mechanism of *B. cinerea* which can be used to develop control strategies against this destructive disease.

Similar resistance mechanisms have been reported in other plant pathogenic fungi. For example, in *Fusarium graminearum*, mutations in the *CYP51* gene, which encodes the target enzyme sterol 14α-demethylase, reduce sensitivity to triazole fungicides like tebuconazole (Liu et al., 2011). Resistance is often accompanied by over-expression of *CYP51* and associated efflux pumps. In *Zymoseptoria tritici*, genome-wide association studies have identified single nucleotide polymorphisms (SNPs) and chromosomonal rearrangements linked to fungicide resistance, particularly affecting genes related to detoxification and membrane transport (Brunner et al., 2016). Among Oomycetes, *Phytophthora infestans* exhibits resistance to metalaxyl, a phenylamide fungicide, primarily due to mutations in genes like *RPA190*, which encodes an RNA polymerase subunit (Gisi and Cohen, 1996). Genomic variations such as gene duplications, deletions, and transposable element insertions have been implicated in the rapid adaptability of such pathogens. Many phyto-pathogens have evolved strategies to evade recognition by plant resistance (*R*) proteins. This includes the deletion, mutation, or epigenetic silencing of avirulence (*Avr*) genes, leading to the breakdown of host resistance. *AVR* genes are highly variable and the variable AVR protein escapes from the recognition mediated by cognate R protein, resulting in breakdown of the resistance function of the *R* gene (Deng et al., 2017; Hu et al., 2022). According to the gene-for-gene paradigm, the recognition of the *AVR* protein by the *R* gene triggers resistance in host plants. Eight *AVR* genes, namely, *AVR-Pi9, AVR-Pita1, AVR-Pia, AVR-Pi54, AVR-Pizt, AVR-Pik*, *AVR-Pii* and *AVR-Pib* in 383 isolates of *Magnaporthe oryzae* in the Sichuan Basin were analyzed by Hu et al. (2022). They discovered that the *AVR* genes of *M. oryzae* employed a variety of strategies, such as short sequence insertion, transposon insertion, gene duplication, nucleotide deletion and substitution, and gene loss, to evade recognition by host *R* genes for pathogenesis. Effectors with suppressive activity have also been reported for *Fusarium oxysporum* f.sp. *lycopersici*, in which the Avr1 effector suppresses resistance by the tomato *R* genes *I‐2* and *I‐3* in response to corresponding effectors *Avr2* and *Avr3*, respectively (Houterman et al., 2008).

Bacterial pathogens also exhibit diverse resistance strategies. *Xanthomonas* spp. and *Erwinia amylovora* have acquired plasmid-encoded resistance genes such as *copA* and *copB* that confer tolerance to copper-based bactericides (Cooksey, 1994; Sundin and Wang, 2018). These genes facilitate the efflux or sequestration of toxic metal ions. *Ralstonia solanacearum*, another significant bacterial pathogen, demonstrates resistance to antibiotics such as streptomycin through ribosomonal mutations and enzymatic inactivation mechanisms (Hayward, 1991). Resistance also manifests in response to plant-derived defenses. For example, in *Leptosphaeria maculans*, loss or repression of the *AvrLm1* gene enables evasion of recognition by *Rlm1-*containing canola cultivars (Gout et al., 2006). The *AvrBST* gene of the bacterial pathogen *Xanthomonas campestris* pv. *vesicatoria* suppresses the activity of *AvrBs1*, an effector gene which triggers hypersensitive response (HR) in host plants (Szczesny et al., 2010).

Understanding how these pathogens evade host defenses enhances disease diagnosis and surveillance, and also provides useful biological information for the development of control strategies. Genomic tools detect resistant plant cultivars early enough, enabling proactive measures. Also, breeding for resistance helps to incorporate broad-spectrum resistance in plants to delay pathogen adaptation. Advancements in CRISPR/Cas9 genome editing and RNAi also directly tackle pathogen resistance mechanisms (Ali et al., 2015; Zhang et al., 2020). Plant pathogen resistance mechanisms are diverse and complex. In the face of emerging pathogenic threats, the use of genomics, transcriptomics, and functional genetics will provide new ways for understanding and managing plant pathogen resistance.

## 4 Understanding the plant-pathogen patho-systems and their role in disease management

Plant pathogens interact with a variety of organisms, such as plants, insects, endophytes and other pathogens. In agro ecosystems and natural biosystems, disease impact on different plants, and evolution and spread of phyto-pathogens remain unclear (Hamim et al., 2024). Plant pathogen spread and diversity can significantly hinder pathogen diagnosis and management efforts (Rubio et al., 2020). Effectors from various pathogens employ different strategies in order to infect host plants. The main mechanisms of effectors in plant-pathogen interactions include destroying the physical barriers of host plants, concealing or defending themselves, establishing favorable conditions for infestation, manipulating the immune responses of plants, and interfering with the physiological activity of host cells (Zhang et al., 2022). For example, root-knot nematodes employ progenitor cell, which induce cell enlargement and continual mitosis without cytokinesis, and finally form a giant cell (Jagdale et al., 2021). This helps in successful parasitism. Also, secretions from the cyst nematode, *Heterodera schachtii*, and the root-knot nematode, *Meloidogyne incognita*, have been found to contain auxin (Oosterbeek et al., 2021). Plant peptide hormone (PPH) mimics facilitate pathogen parasitism, and multiple classes of PPH effector mimics, including C-terminally encoded peptide (CEP)-like, inflorescence deficient in abscission (IDA)-like, and clavata3/embryo surrounding region (CLE)-like peptides, have been recorded in nematodes (Ronald and Joe, 2018). Another example is *Pseudomonas syringae pv. tomato* strain DC3000 (*Pto* DC3000) which manipulates auxin signaling to alter root development and can gain entry into its host through wounds caused by emerging lateral roots (Kong et al., 2020). *Pto* DC3000-induced lateral root formation is facilitated by auxin response factor 7 (ARF7) and ARF19. SA, an important phytohormone that acts against pathogens, can block bacteria from entering into plants by suppressing lateral root formation. ARF7 antagonizes SA marker genes expression, thereby promoting lateral root development (Kong et al., 2020).

Plant pathologists study the biology of plant pathogens in order to find ways through which disease control measures can be developed. Endophytic fungi are utilized as the most common microbial biological control agents (MBCAs) against phytopathogens. They produce a range of antifungal secondary metabolites such as enzymes, antibiotics, and lipopeptides through colonization, and compete with other pathogenic microorganisms for nutrients and space (Akram et al., 2023). Virulence mechanism of emerging pathogens is usually difficult to detect. Genomic data can provide the information required to identify the possible toxins and effectors possessed by the pathogen. The main impacts of genome sequences in plant pathology are improved knowledge of the pathogenicity, genome evolution, and life-style of pathogens (Aylward et al., 2017).

### 4.1 Mechanism of host susceptibility and resistance

Understanding the molecular basis of host-pathogen interactions is key to developing disease-resistant crops and enhancing agricultural productivity (Ijaz et al., 2024). These interactions can be analyzed from two perspectives: identifying the virulence strategies of pathogens and deciphering host defense mechanisms (De Moraes Pontes et al., 2020). Plants have developed a complex immune system in order to defend themselves against any threat from pathogens. They use their innate immunity, made up of intracellular and cell surface receptors, to detect the molecular signatures (pathogen-associated molecular patterns[PAMPs]) used by pathogens to infect plants. PAMP-triggered immunity (PTI) effectively defends plants from a wide range of pathogens. Though, pathogens can subdue the host plant immune response by secreting their small proteins (effectors) into the host cell cytoplasm. When PTI is no longer effective due to the effect of pathogenic effectors, effector-triggered immunity (ETI) is activated to provide resistance to the host (Nguyen et al., 2021). Susceptibility or resistance of plants is determined by the interaction between effectors and the plant immune network. The plant immune system is a dynamic system that keeps evolving to defend plants against environmental threats Bhadauria and Zhao (2024). An overview of the two types of host immunity in plants is presented in **Table 2**.

**Table 2:** Overview of the differences between the two host resistance mechanisms in plants.

Characteristic	Pattern-triggered immunity (PTI)	Effector-triggered immunity (ETI)	References
Order of defense	First line of defense	Second line of defense	Hou et al., 2019; Nguyen et al., 2021
Immunity trigger in pathogen	Conserved pathogen-associated molecular patterns (PAMPs)	pathogen-specific elicitors (effectors)	Bent and Mackey, 2007
Immunity activators in plant	Pattern recognition receptors (PRRs)	plant resistance (*R*) gene products or Intracellular resistance proteins (*R*) e.g., nucleotide-binding leucine-rich repeat receptors (NLRs)	Hou et al., 2019; Cui et al., 2015
Location of immunity on-set	Plasma membrane (extracellular)	Cytoplasm or nucleus (intracellular)	Hou et al., 2019
Defense response	Production of reactive oxygen species (ROS), influx of extracellular Ca^2+^ into the cytosol, activation of mitogen-activated protein kinases, etc.	Rapid production of ROS, hypersensitive response, systemic acquired resistance (SAR)	Nomura et al., 2012; Zhang et al., 2012; Hou et al., 2019; Nguyen et al., 2021
Purpose	Restriction of pathogen growth	Complete resistance of pathogen (subsequent death of pathogen)	Nguyen et al., 2021
Pathogen strategy	Plant may become susceptible to pathogen through effector-triggered susceptibility (ETS) when pathogens release their effectors.	Pathogens may produce new effectors	Nguyen et al., 2021

Plant defense compounds which can be used as organic pesticides can be successfully identified and characterized through genomics. The genes responsible for the production of these compounds can be incorporated into other varieties of the same crop or other crop species to increase resistance. Also, important genes in pathogens (such as virulent genes, genes responsible for growth and development, and immunity) can be identified and suppressed or silenced in order to confer resistance on the host. *Puccinia striiformis* f.sp. tritici (Pst) is responsible for stripe rust disease of wheat (Chen, 2020). Wheat plants have developed complex defense mechanisms to combat Pst., using such mechanisms as hypersensitive response (HR) and programmed cell death (PCD). Transcription factors (TFs) play major roles in plant defense response. *TaMYB391*, a Myeloblastosis (MYB) transition factor (R2R3 MYB TF) identified through real-time PCR (RT-qPCR), was shown to be up-regulated during Pst infection and was associated with HR-related gene expression through salicylic acid signaling. RNAi-mediated silencing of *TaMYB391* resulted in increased susceptibility to Pst. confirming its role in resistance (Wang et al., 2019; Bia et al., 2021; Hawku et al., 2022).

Oil palm (*Elaeis guineensis*) is threatened by the fungus, *Ganoderma boninense*, which causes, basal stem rot (BSR). This disease can make an oil palm plantation to lose 43% of its economic value in just six months (Khoo and Chong, 2023). Tee et al. (2013) discovered several genes in oil palm related to defense against G. *boninense*. RNA-sequencing (RNA-seq) identified seven differentially expressed genes (DEGs) involved in the defense response, including putative senescence-associated protein, and thaumatin-like protein. These genes can serve as biomarkers to detect BSR in oil palms at the early stage of infection (Zuhar et al., 2021). Infection starts at the epidermal surface of the root tissue and progresses to the xylem vessels (Alexander et al., 2017). Recognition process begins when PAMPs such as glucans, ergosterol, and chitin, from *G. boninense* binds to pattern recognition receptors (PRRs) of the host (Ho et al., 2016).

Resistance (R) proteins in the host can directly or indirectly detect these pathogen effectors. Genome-wide association studies (GWAS) can identify R genes by analyzing populations of resistant and susceptible plants. High-throughput sequencing (HTS) has uncovered numerous R genes, including the NLR (nucleotide-binding leucine-rich repeat) gene family, which are instrumental in disease-resistance breeding (Dalio et al., 2017; Hamim et al., 2022). A GWAS in citrus predicted over 508 nucleotide-binding site (NBS) genes as potential R genes (Wang et al., 2015).

**Figure 3:**
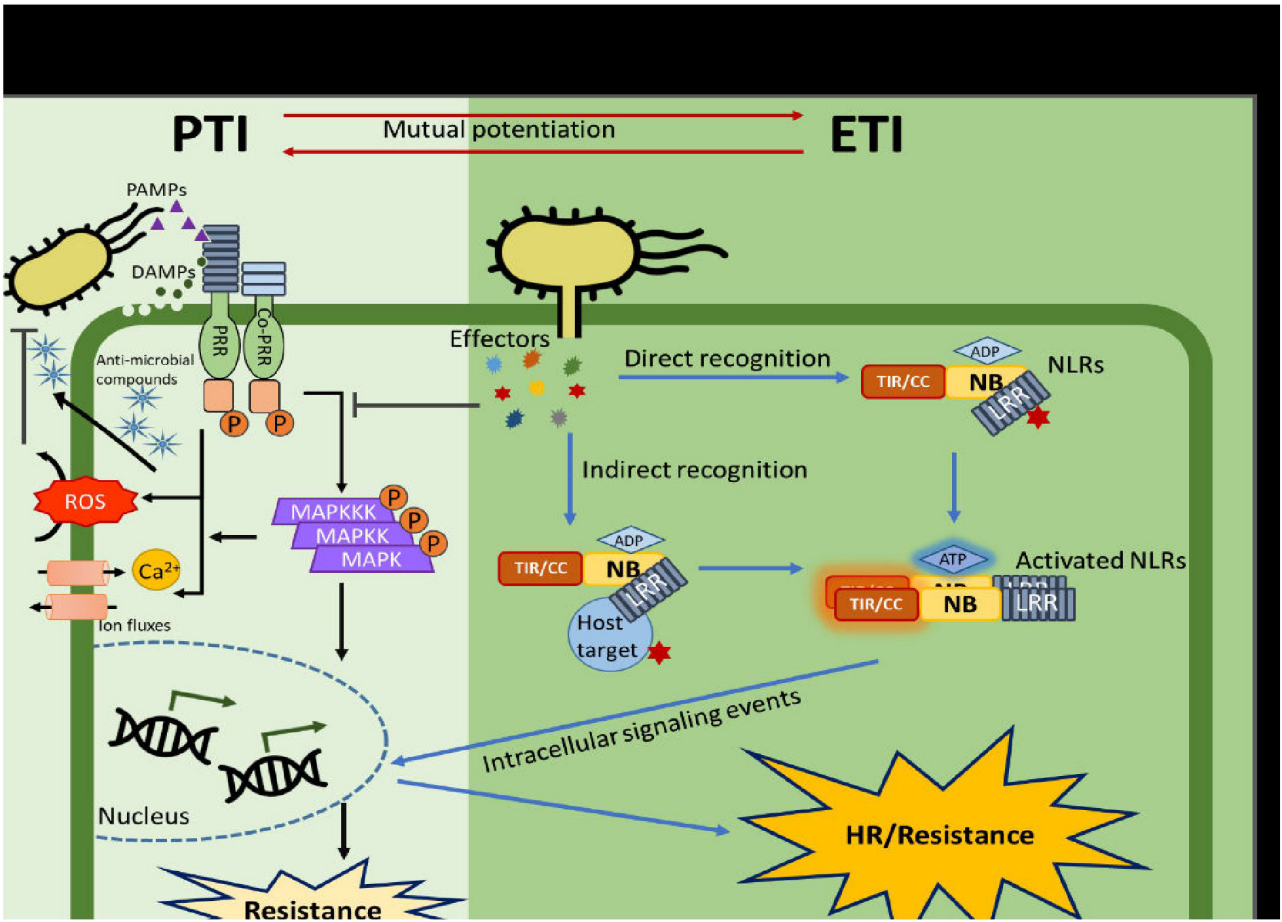
Schematic view of pattern-triggered immunity (PTI) and effector-triggered immunity (ETI) in plants (Adapted from Nguyen et al., 2021).

Common or specific effectors of phyto-pathogens, which are useful in the detection of host susceptibility mechanism, can be identified from genomic and transcriptomic data. For instance, comparative genomics of *Xanthomonas campestri* pv. campestri (Xcc), responsible for citrus canker disease, identified pthA4, a key effector that activates the host *S* gene, *CsLOB1*. This gene is targeted by a wide range of TAL effectors (Hu et al., 2014). Another example is the whitefly-mediated transmission of cotton leaf curl virus (*CLCuMuV*). Two whitefly species (MEAM1 and Asia II 7) vary in their capability to effectively transmit cotton leaf curl Multan virus (*CLCuMuV*). MEAM1 is a poor vector of *CLCuMuV* while Asia II 7 is an efficient vector. Through RNA interference, yeast two-hybrid system, bimolecular fluorescence complementation, RT-qPCR, bioassays and bioinformatics, the interaction between a whitefly (*Bemisia tabaci* Asia II 7) innate immunity-related protein (*BTB/POZ*), and a Cotton leaf curl Multan virus (*CLCuMuV*) protein (viral *AV1* [coat protein]) was detected (Farooq et al., 2022). The virus inhibits the innate anti-viral immunity of the whitefly vector by significantly suppressing *BTB*/*POZ* transcription and some anti-viral immune signaling pathways (*Jak*/*STAT*, *Toll*, *Jnk* and *Imd*) in order to enhance the accumulation of *CLCuMuV* in Asia II 7. *CLCuMuV* is primarily a pathogen of cotton but also attacks other plants such as those in the Malvaceae family (*Hibiscus esculentus*, *H. rosa-sinensis*, *H. Cannabinus*, *Gossypium hirsutum* and *Malvaiscus arboreus*) (Du et al., 2015; Chen et al., 2019). A Schematic view of pattern-triggered immunity (PTI) and effector-triggered immunity (ETI) in plants is presented in **Figure 3**.

Our knowledge of the interactions between host plants and their pathogens as well as the gaps in plant response to biotic stressors will be improved by the data from genomics research. These data offer a framework for creating long-term plans for managing economically important diseases. This information will accelerate crop improvement, which will be crucial for increasing agricultural productivity for global food security (Panwar and Bakkeren, 2024).

## 5 Genomic approaches for controlling plant pathogens

Advancements in genomics have revolutionized plant disease management by providing precise tools for identifying and controlling phytopathogens. High-throughput sequencing (HTS) plays a vital role in accurately detecting pathogens, enabling the formulation of effective, long-lasting strategies for disease prevention and control (Piombo et al., 2021). Innate defense mechanisms are triggered by pathogen recognition and invasion in plants. The detection of invasive pathogens, activation of defensive pathways, and signal transduction are part of the coordinated steps that make up the plant immune response. In order to evade plant immunity, pathogens have developed a variety of structures. Based on these facts, genetic improvements in plants are necessary for long-term disease control and prevention in order to guarantee global food security. Modern techniques such as gene editing have emerged as powerful tools for enhancing plant resistance. Targeting *S* genes using CRISPR technologies has opened new frontiers in disrupting the molecular compatibility between hosts and pathogens (Ijaz et al., 2024).

Genome editing techniques such as CRISPR/Cas9, CRISPR/Cas13, ZFNs, TALENs and base editing, have been successfully used to improve disease resistance in crops through several methods including gene knockdowns, knockouts, modifications, targeted mutagenesis and activation of target genes (Manzoor et al., 2024). Among these techniques, CRISPR/Cas9 stands out due to its exceptional effectiveness, low chance of off-target effects, and simplicity of application. CRISPR-mediated disease-resistant crops are developed to target host’s *S* gene and *R* gene, and pathogen effectors that inhibit their development, and broad-spectrum disease resistance (Manzoor et al., 2024).

### 5.1 Clustered regularly interspaced short palindromic repeats

Through genome sequencing, researchers can identify *R*-genes and their interaction with avirulence (*Avr*) genes (Silva et al., 2019), facilitating the development of rapid and reliable diagnostic tools and improved disease-resistant cultivars (Klosterman et al., 2016). Various potential R genes from different plants can be easily identified and isolated using physical mapping. Bioinformatics tools have been used to identify and analyze a vast number of genetic variations, such as disease-causing mutations in the genome. These mutations can be utilized in the development of disease resistant cultivars (Joshi et al., 2023). Bioinformatics tools or reference genome data are used for R–Avr co-localization studies. Kunz et al. (2025) developed an assay, the AD assay, which identifies candidate genes with high accuracy, reduced time and cost when compared to previous approaches. The authors deployed the assay to identify *AvrPm60*, the avirulence effector, recognized by *Pm60*, the wheat immune receptor, and characterized the requirements of *Pm60*-mediated resistance. This can guide future breeding decisions. Arndell et al. (2024) developed a platform for the rapid identification of *R*–*Avr* gene pairs. They isolated novel and known *Avr* genes from wheat stem rust pathogen, *Puccinia graminis* f. sp. *tritici*. Timely *Avr* gene identification offers molecular tools to understand and track pathogen virulence evolution through genotype surveillance. CRISPR/CaS9, a type of adaptive immunity, has been developed as a gene editing tool and has been applied on over 20 plants (Ricroch et al., 2017). CRISPR/Cas9 has been extensively used in genome editing because it is easier to develop and more affordable compared to other gene editing methods like TALEN and Zinc Finger since it leverages the bacterial cellular machinery to modify DNA. It integrates the virus sequence into the CRISPR locus and transcribes small RNAs that direct Cas-9 endonuclease to target and cleave the sequence of the invading pathogen (Doudna and Charpentier, 2014).

CRISPR/Cas9 has been utilized to interfere with strigolactone (SL) biosynthesis in *Oryza sativa* (rice) and *Lycopersicon esculentum* (tomato) by editing the CCD7 and CCD8 genes respectively, reducing germination of the parasitic weeds, *Striga hermonthica* and *Phelipanche aegyptiaca* (Butt et al., 2018; Bari et al., 2019). This demonstrates the potential of genome editing in managing complex parasitic interactions in crops. Resistance provided by the deletion of an *S* gene is considered to be more robust than *R* gene-mediated resistance since it is not based on the detection of effectors that can rapidly evolve and bypass *R* gene-mediated resistance (Gorash et al., 2021).

### 5.2 RNA interference

The two main levels at which gene silencing operate in plants and a wide variety of other eukaryotic systems are: transcriptional gene silencing (TGS) mechanisms, which modify DNA and chromatin to change the rate of transcription, and post-transcriptional gene silencing (PTGS) mechanisms, which breaks down homologous RNA molecules and/or prevent their translation (Baulcombe, 2004). PTGS is a cellular process that subjects double-stranded RNA (dsRNA) molecules to degradation after they have been transcribed (Ashfaq et al., 2020), while TGS is a nuclear-localized mechanism that inhibits transcription by preventing transcriptional machinery from binding through the blockage of a promoter region (Vaucheret et al., 2001). Transposable elements (TEs) which are auto-replicative short DNA repeats that can change position within the genome (Sun et al., 2015), are suppressed by TGS. The process commences with the reduction of the protein levels and mRNA of active TEs by PTGS and small interfering (si) RNAs. This facilitates the onset of TGS activity by regulating the first deposition of DNA methylation (Trasser et al., 2024). Plants export endogenous small RNAs (sRNAs) into their pathogen counterparts to knockdown certain genes that are important in pathogenesis. This mechanism is said to be bi-directional as fungal sRNA effectors target plant genes as well. This process is called cross-kingdom RNAi (Mann et al., 2023). Weiberg et al. (2013) reported the movement of pathogen sRNA into the host plant in *Botrytis cinerea* infection of tomato and Arapidopsis (*Arabidopsis thaliana*). The authors detected the transportation of transposon-derived sRNAs (*Bc-sRNAs*) of *B. cinerea* into the plant hosts in order to collapse the host-encoded immunity genes.

RNA interference (RNAi) has been used to enhance resistance against several plant pathogens on crops. RNAi can be conducted through three different approaches viz, host-induced gene silencing (HIGS), virus-induced gene silencing (VIGS), and spray-induced gene silencing (SIGS) (Panwar et al., 2016, 2017). The working principle of these technologies is to silence targeted genes in pathogens or pests. Ibanez et al. (2023) used a VIGS-based RNAi approach to reduce the growth and reproductive ability of the insect, *Diaphorina citri*, a vector of the bacteria, *Candidatus* Liberibacter asiaticus (CLas) and *Candidatus* Liberibacter americanus (CLam), which are phloem-inhabiting pathogens of citrus responsible for Huanglongbing (HLB), the most significant and destructive citrus disease in the world. In the study, a CTV-based double stranded RNA (CTV-dsRNA) was introduced into *Citrus macrophylla* plants through the bacteria vector, *D. citri*. The CTV-dsRNA targeted the *DCcathL* and *DCcathB* genes in *D. citri* which are important genes involved in digestion, immunity, embryogenesis, and ecdysis of the insect. The study successfully reduced vector population.

Crop protection based on HIGS is an example of cross-kingdom RNAi and involves the absorption of dsRNA and/or sRNA derived from transgenes into the pathogen to prompt silencing of a gene that plays an important role in virulence. This has been applied to some fungal and viral plant diseases (Koch et al., 2013; Koch and Wassenegger, 2021). It has been demonstrated that in *Gossypium hirsutum* (cotton) plants infected by *Verticillium deahliae*, there is an increase in the levels of two highly conserved, cotton-encoded miRNAs (miR159 and miR166) that target pathogens following infection. These miRNAs are then transported into the fungus to down-regulate two important virulence genes of the pathogen: *HiC-15* and *Clp-1* (Zhang et al., 2016).

SIGS circumvents the drawbacks of using genetically modified (GM) crops. It involves direct application of dsRNAs or siRNAs onto plant surfaces. It is especially useful against foliar fungal infections and emerging pathogens due to its broad applicability and efficiency (Degnan et al., 2022; Niño-Sánchez et al., 2022). SIGS can be used on any crop species and has an added advantage of being very effective at controlling emerging pathogens. To control fungal pathogens that attack aerial parts of plants such as fruits and vegetables, SIGS is highly recommended (Mann et al., 2023).

### 5.3 Genomic insights into biological control potentials

Through genomic studies, researchers can identify the genes responsible for the promotion of plant growth, production of antibiotics, and inhibition of plant pathogens. Bio-control is the use of natural antagonists, like fungi and bacteria in place of synthetic pesticides, which are not eco-friendly. Various bacterial genera such as *Agrobacterium*, *Acinetobacter*, *Bacillus*, *Pseudomonas*, *Streptomyces*, *Paenibacillus*, *Azotobacter*, *Bradyrhizobium*, *Azospirillum* and *Rhizobium* are widely recognized for their bio-control potentials (Massawe et al., 2018; Ayaz et al., 2021; Zubair et al., 2021). These bacteria inhibit pathogens by competing for nutrients, producing antimicrobials, and inducing systemic resistance (Farzand et al., 2019; Ayaz et al., 2022; Lee et al., 2023). Fungal bio-control agents such as *Trichoderma*, *Penicillium*, and *Aspergillus* also play significant roles in suppressing plant diseases (Thambugala et al., 2020). They produce secondary metabolites like alkaloids, terpenoids, and isocoumarins with wide-ranging pesticidal activities (Contreras-Cornejo et al., 2016; Alam et al., 2021; Risoli et al., 2022).

Ma et al. (2023) studied the rhizosphere bacteriome of tobacco infected by black shank disease using illumina sequencing. The phylum Actinobacteria was enriched in the diseased samples in comparison to the other samples. In soils treated with the bio-control agent, *Bacillus velezensis* S719, the genera *Sphingomonas* and *Bacillus* were significantly increased. These organisms contribute to disease suppression through antagonistic activities, biofilm formation and enhanced immune signaling (Asaf et al., 2020; Guo et al., 2020; Sun et al., 2022b).

Gattoni et al. (2023) demonstrated the suppression of *Meloidogyne incognita* by *Bacillus firmus* I-1582 and *B. amyloliquefaciens* QST713 using split root tests, RT-qPCR, and qPCR. These bacteria reduced population density of *M. incognita* by increasing the mortality rate by more than 75%. They activated a short-term defense response (24 h), by boosting the signaling of an intermediate jasmonate, and a long-term defense response through salicylic acid pathways. These two organisms can therefore be employed in the development of integrated pest management strategies.

## 6 Development of genomics tools for rapid and early pathogen identification

Advances in genomics have made it possible to rapidly characterize plant pathogen genomes and discover characteristics that make it easier to understand the biology of phyto-pathogens. This has led to a deeper understanding of pathogens’ genetics, aiding in the development of diagnostic tools for early pathogen detection and control of emerging diseases (Aylward et al., 2017). Comparative genomics has been used to identify conserved regions that can be used as basis for the diagnosis of diseases caused by *Pseudoperonospora cubensis* (Withers et al., 2016) and two species of *Calonectria* (Malapi-Wight et al., 2016). These approaches improve quarantine measures by enabling early detection, identification and suppression of diseases (McTaggart et al., 2016). Various rapid field diagnostic instruments have been developed, allowing for real-time detection of pathogens (Kannan and Bastas, 2015). These technologies support on-site analysis within hours of sample collection, drastically reducing diagnosis time. An example is bacterial etiolation and decline (BED) of creeping bentgrass, caused by *Acidovorax avenae*, a frequently misdiagnosed disease. Giordano et al. (2018) developed a sensitive and specific real-time PCR assay for detecting pathogenic *A. avenae* using 0017 and 0019 primer sets with ZEN probes. The assay detects the pathogen directly from infected turfgrass within five hours, enabling quick and accurate field diagnosis, thus reducing misdiagnoses and unnecessary fungicide application.

The advent of third-generation sequencing technologies has also improved plant disease diagnosis. The ONT device offers significant benefits for in-field diagnosis because of its portability, rapid sequencing, and simplified sample preparation (Mehetre et al., 2021). Boykin et al. (2019) demonstrated this in their cassava virus action project (“Tree Lab”), which utilized MinION and MinIT mobile sequencing devices for field-based extraction, sequencing, and diagnosis of cassava mosaic begomoviruses across Sub-Saharan Africa (Nigeria, Kenya, Uganda, and Tanzania). Entire workflows, from sampling to result interpretation, were completed within three hours, eliminating the need for central laboratories.

Biosensors are analytical devices composed of a bio-recognition element and a physicochemical transducer that convert a biological reaction into a detectable electrical signal upon binding of the target analyte (Hameed et al., 2018; Bridle and Desmulliez, 2021). These sensors also rely on optical, chemical, electrochemical, vibrational or magnetic signals (Tiwari et al., 2017). Biosensors are good point-of-care tools because they are typically inexpensive, easy to use, and can produce results quickly (Bridle and Desmulliez, 2021). Li et al. (2019b) developed a smart phone-based VOC (volatile organic compound) biosensor for the detection of late blight disease in tomato leaves. The sensor used cysteine functionalized gold nanoparticles, which change color upon VOC exposure. A nanoparticle-coated strip is inserted into a device connected to a pump, drawing air across the strip. The resultant colorimetric change can then be analyzed using the smart phone camera. This device has been tested and yielded positive results for both field-collected infected leaves and artificially inoculated tomato leaves.

## 7 Challenges of genomics in the identification and control of phyto-pathogens and the way forward

Despite these advancements, several limitations remain in applying next-generation sequencing (NGS) and metagenomics for pathogen identification (Nezhad, 2014). PacBio and MinION have very high error rates (10 to 15% per base), but this has slightly improved over the years. For PacBio platform, DNA molecules are sequenced many times, and this reduces the error rate to a minimum of 0.1% at a rate of 9 to 11 times (Tedersoo et al., 2017). Sequencing *in situ* often yields mixed DNA from hosts and other microbes, complicating pathogen genome assembly. Low pathogen abundance further impairs DNA/RNA yield for sequencing. To address these issues, numerous targeted enrichment techniques, such as DNA or RNA hybridization and sequence capture, PCR amplification, and cell enrichment have been extensively used in conjunction with NGS sequencing (Cronn et al., 2012). Other factors hindering the broader use of NGS include high costs, technical complexity, and data interpretation challenges (Vashisht et al., 2023; Islam, 2024). The cost of NGS is significantly higher than that of conventional diagnostic methods. Unlike PCR or serological tests, NGS involves multiple molecular techniques, extended timelines, and computational tools—many of which require bioinformatics expertise Also, majority of the free data analysis software require some computational programming skills. Software with graphical user interface may have high licensing and support fees. Nonetheless, NGS remains invaluable for large-scale pathogen surveillance (Díaz-Cruz et al., 2019), especially with ongoing interdisciplinary and international collaborations. In spite of high error rate, the nanopore technology has shown great success in disease diagnosis due to less analysis time and low cost of equipment (Quick et al., 2016). The issues associated with NGS can be resolved to enhance the use of genomics in plant pathogen identification through multi-disciplinary and international collaborations, better knowledge of microbial ecology, constant training of researchers to obtain adequate skilled personnel, management of technical and logistic barriers, advancements in sequencing technologies and improved data analysis and interpretation (Vashisht et al., 2023; Nizamani et al., 2023; Islam, 2024).

Metagenomic assembly presents significant technical and computational challenges due to the complexity and diversity of microbial communities in environmental samples. Unlike genome assembly for a single organism, metagenomic assembly requires the reconstruction of multiple genomes simultaneously from a mixture of DNA sequences representing many species with variable abundances. This complexity demands sophisticated data analysis platforms and substantial computational resources, particularly high memory and processing power, to handle the massive volume of sequencing reads and the intricate assembly generated (Lapidus and Korobeynikov, 2021; Ayling et al., 2020). A major hurdle in metagenomic assembly is the difficulty in detecting sequence overlaps and accurately assembling contigs for closely related species or strains because their genomes can be highly similar. This similarity complicates the assembly by creating ambiguous branching paths that assemblers struggle to resolve, often resulting in fragmented or chimeric contigs. Additionally, species present in low abundance frequently have insufficient sequencing coverage, which limits the ability to assemble large and contiguous genomic segments, further fragmenting assemblies for rare taxa (Lapidus and Korobeynikov, 2021). Another challenge is the limited availability and completeness of reference databases for taxonomic and functional classification of assembled contigs. This is especially problematic for poorly characterized groups such as many fungi and uncultured microorganisms, where reference genomes are scarce or absent, leading to difficulties in accurate detection and annotation (Ayling et al., 2020; Piombo et al., 2021). Moreover, metagenomic datasets tend to produce false-positive taxonomic assignments because sequences from universally conserved regions can incorrectly map to multiple species, confounding microbial community profiling. The choice of sequencing technology also influences assembly outcomes. Widely-used second-generation platforms like Illumina, which have enabled widespread metagenomic studies, produce short reads. However, these short reads limit the ability to resolve long repetitive genomic regions and structural variations, often resulting in highly fragmented draft assemblies that complicate downstream comparative and functional analyses (Land et al., 2015). *De novo* assembly using short reads struggles to accurately reconstruct genomes with repetitive elements larger than the read length, producing assemblies with gaps and ambiguities. Conversely, third-generation long-read sequencing technologies, such as Oxford Nanopore MinION and Pacific Biosciences (PacBio) single-molecule real-time sequencing, generate reads that span thousands to tens of thousands of bases (Li et al., 2016). These long reads can bridge repetitive regions and complex genomic structures, substantially improving the completeness of assembled genomes. Their integration in metagenomic workflows facilitates near-complete genome assembly and enhances resolution of strain-level diversity, although error rates and cost considerations remain factors to balance (Quick et al., 2014; Brown et al., 2014; Amarasinghe et al., 2020).

The main disadvantage of HIGS technique is the substantial challenges associated with permanently altering crop species, such as the high cost, extended development periods, and degree of difficulty in producing genetically modified variants of every crop species. Therefore, HIGS is ineffective in the case of a new pathogen colonizing a plant that has already being genetically modified. On the other hand, SIGS has its own disadvantages. The main factors impeding the technique are: short half-life of the applied RNA molecules, limited RNA uptake into the host plant and pathogen, and movement of the RNA biopesticide to all sites of infection, especially the underground parts, including roots, tubers, bulbs, rhizomes, and corms. Solving these challenges is crucial and will aid future applications of SIGS technology in field studies (Mann et al., 2023).

## 8 Discussion

The first and most vital step in plant disease control is the early and accurate identification of pathogens. Early detection of causative agent of a disease enables prompt development of appropriate management strategies to reduce crop losses (Khakimov et al., 2022). Traditional techniques for detecting plant pathogens have long been the cornerstone of plant disease diagnostics. However, these methods are often time-consuming, labor-intensive, and require significant expertise. A major challenge with traditional diagnostics is the risk of misdiagnosis, especially when disease symptoms are interpreted by less experienced observers. Many plant diseases manifest overlapping or non-specific symptoms, increasing the chance of incorrect identification and leading to inappropriate management strategies (Trippa et al., 2023). To address these limitations and improve the speed and accuracy of pathogen detection, a range of new molecular and genomic technologies have been developed in recent years. These modern methods not only accelerate diagnostic workflows but also enhance precision by relying on the detection of pathogen-specific genetic markers rather than solely on visible symptoms or culturing (Khakimov et al., 2022). Genomics, in particular, has emerged as a powerful tool in plant pathology, revolutionizing the study of pathogens that are difficult or impossible to culture using traditional microbiological techniques. Culture-independent genomic approaches enable direct analysis of microbial DNA and RNA extracted from infected plant tissues, circumventing the time and biases associated with cultivation. By sequencing pathogen genomes or metagenomes, researchers can identify major genes involved in virulence, pathogenicity, and host adaptation, providing comprehensive insights that were previously inaccessible (Bard et al., 2024; Taliadoros and Stukenbrock, 2023). NGS technologies can be used to rapidly diagnose diseases and identify emerging pathogens, and also non- cultivable pathogens which would ensure the complete characterization of all the microorganisms present in a plant sample (microbiome) (Aragona et al., 2022). NGS can sequence millions of DNA fragments at once, providing comprehensive information about genomes structure, variations, activities, and behavior changes of genes. Advancements in sequencing have focused on faster, more accurate, relatively cheap and highly improved sequencing technologies, and this has been achieved with NGS.

Various genomic approaches have been successfully applied to manage plant pathogenic diseases. Sequenced genomes of plant pathogens offer blueprints that can be used to predict several strategies that pathogens employ to infect plants and also identify potential virulent factors possessed by these pathogens. Virulent and avirulent strains of pathogens, or strains from various hosts and regions, can be compared to investigate the virulence mechanisms for plant pathogens (Xu and Wang, 2019). Integrating transcriptomic, proteomic, and genomic data allows for the identification of novel peptides and important virulence factors, the improvement of existing gene models, and the investigation of host system responses and changes (Xu and Wang, 2019). Plant disease is an active and dynamic process; the physiological state of pathogens and their hosts will alter as disease progresses. Our understanding of pathogen biology and the complex interactions between pathogens and their host plants has significantly improved with the advent of high through-put sequencing (Singh et al., 2023). Plant host responses during disease development have been extensively studied using RNA-sequencing (RNA-seq) technology. Through RNA sequencing and microarray techniques, citrus physiological changes caused by *Candidatus* Liberacter asiaticus has been recorded (Rawat et al., 2015; Yu et al., 2017).

New developments in genome editing technology provide remarkable pathways to improve plant resistance against infections by targeting susceptibility genes, which pathogens use to initiate infection and replicate within the host plant (Rani et al., 2024). Genomic tools such as draft genome sequence of pathogens and their host plants, and the gene editing tool, CRISPR-cas9 offer researchers the opportunity to discover and incorporate resistance in host plants to increase plant yield (Silva et al., 2019). RNA interference (RNAi) has shown success in enhancing resistance against a broad range of phyto-pathogens in a variety of crops through its approaches such as host-induced gene silencing (HIGS), virus-induced gene silencing (VIGS), and spray-induced gene silencing (SIGS) (Panwar et al., 2016, 2017). Some fungal and bacterial organisms have also been reported to have bio-control potentials; they secrete compounds and contain genes that can inhibit plant pathogens. The detection of several secondary metabolites through genomics, such as steroids, terpenoids, quinines, alkaloids, peptides, benzopyranones, and isocoumarins from these organisms provided the foundation for the development of agrochemicals with potential anti-fungal, herbicidal, anti-bacterial, insecticidal, nematicidal, and other agricultural applications (Contreras-Cornejo et al., 2016; Risoli et al., 2022).

Monitoring for early detection of plant diseases is essential in mitigating the risks posed by plant pathogens on food security (Johnson et al., 2023). Genomic surveillance is a significant tool for the diagnosis, early detection, and management of emerging plant diseases. The approach offers molecular insights into the interactions between pathogens and their host plants, which is crucial for the development of effective disease management strategies. Genomic surveillance through techniques such as metagenomics, enables the detection of emerging threats, monitoring of pathogen evolution, and prediction of disease outbreaks (Islam, 2024). Biosensors and portable devices have been developed and commercialized, and they offer on-site diagnosis of diseases, while advances in bioinformatics have improved analysis of complex datasets (Prasanna et al., 2024).

## 9 Conclusion

Early detection of plant pathogens is crucial in plant pathology, as it helps minimize agricultural losses. Accurate identification of phytopathogens is essential for developing effective management strategies to control these pathogens. Genomics offers a wide array of tools that enable timely detection and precise identification of pathogens. However, no single genomic method is universally perfect; the choice of detection technique depends on factors such as cost, the bioinformatics expertise of users, the time required for analysis, and the availability of tools in a given region. While some techniques are time-consuming, others provide rapid and reliable results. The efficiency of these tools, along with the quality and volume of data they generate, can vary significantly. Recently, point-of-need genomic tools and devices have been developed, advancing genomics to a new level. Wider affordability and accessibility of these technologies have the potential to drastically reduce plant diseases and contribute significantly to global food security in the near future.
